# Clinical Significance of Cytokeratin 19-Fragments (CYFRA 21-1), Osteopontin (OPN) and Human Epididymis Protein 4 (HE4) in Pancreatic Adenocarcinoma

**DOI:** 10.3390/ijms27031562

**Published:** 2026-02-05

**Authors:** Augustin Catalin Dima, Daniel Vasile Balaban, Iulia-Ioana Stanescu-Spinu, Ana Teodorescu, George Manucu, Laura Ioana Coman, Alina Dima, Cezar Betianu, Mihai Tanase, Daniela Miricescu, Mariana Jinga, Catalin Carstoiu

**Affiliations:** 1 Clinical Department 14, Faculty of Medicine, Carol Davila University of Medicine and Pharmacy, 050474 Bucharest, Romania; augustin.dima@drd.umfcd.ro (A.C.D.); catalin.carstoiu@umfcd.ro (C.C.); 2 Department of Surgery 2, Carol Davila University Central Emergency Military Hospital, 010825 Bucharest, Romania; 3 Clinical Department 5, Faculty of Medicine, Carol Davila University of Medicine and Pharmacy, 050474 Bucharest, Romania; alina.dima@umfcd.ro (A.D.); mariana.jinga@umfcd.ro (M.J.); 4 Department of Gastroenterology, Carol Davila Central University Emergency Military Hospital, 010825 Bucharest, Romania; ana-maria.teodorescu@rez.umfcd.ro (A.T.); george.manucu@drd.umfcd.ro (G.M.);; 5 Discipline of Physiology, Faculty of Dentistry, Carol Davila University of Medicine and Pharmacy, 050474 Bucharest, Romania; 6 Department of Radiology, Doctor Carol Davila Central Military University Emergency Hospital, 010825 Bucharest, Romania; cezar.betianu@gmail.com; 7 Discipline of Biochemistry, Faculty of Dentistry, Carol Davila University of Medicine and Pharmacy, 050474 Bucharest, Romania; daniela.miricescu@umfcd.ro; 8 Department of Orthopedics and Traumatology, University Emergency Hospital, 050098 Bucharest, Romania

**Keywords:** CYFRA 21-1, osteopontin, pancreatic cancer, human epididymis protein 4, biomarker

## Abstract

Pancreatic cancer remains one of the most aggressive digestive neoplasms, especially due to late diagnosis. The aim of our study was to investigate cytokeratin-19 fragments (CYFRA 21-1), osteopontin (OPN), and human epididymis protein 4 (HE4) clinical significance in pancreatic adenocarcinoma. Our research is a single-center cross-sectional prospective study that included sixty hospitalized patients diagnosed with pancreatic adenocarcinoma and fourteen controls. CYFRA 21-1, OPN, and HE4 were tested in all participants using Luminex x MAP technology. Serum CYFRA 21-1 levels were weakly correlated with those of OPN (r = 0.302; *p* = 0.009), HE4 (r = 0.485; *p* < 0.001), and carbohydrate antigen (CA) 125 (r = 0.376; *p* = 0.037). Similarly to CA 19-9 and CA 125, the serum OPN levels were higher in patients with pancreatic cancer when compared to controls, 3.37 (1.84; 9.12) ng/mL versus 1.59 (1.09; 2.51) ng/mL; *p* = 0.003. However, in multivariate analysis, the OPN was not an independent predictor for pancreatic cancer. Further, the receiver operating characteristic (ROC) curve analysis identified CA 19-9 as the biomarker with the highest diagnostic accuracy, while CYFRA 21-1, OPN, and HE4 did not reach clinically meaningful results. Further, the CYFRA 21-1 levels were significantly higher in cases subjected to significant weight loss before admission.

## 1. Introduction

Pancreatic cancer remains one of the most aggressive and lethal malignancies, marked by a poor overall prognosis [1,2,3]. Furthermore, pancreatic cancer stands as the fourth-leading cause of cancer-related deaths, with a 5-year overall survival rate of 13% [4]. Despite the significant progress that has been achieved in other cancers, the mortality rate of pancreatic cancer remains high [5,6]. The reported median of the survival time is less than two years for surgically treated patients [2,7].

Pancreatic adenocarcinoma is the most prevalent histological type of pancreatic neoplasia. Due to the rapidly aging population, it is predicted that pancreatic adenocarcinoma will be the second-leading cause of mortality by 2030, particularly within older demographics that exceed 65 years of age. There are no early detection tests, and in the majority of cases, there are neither recognizable clinical symptoms nor signs specific to the early stages of the disease. It is only in advanced disease stages that clinical features like jaundice or abdominal pain become evident.

The carbohydrate antigen 19-9 (CA 19-9) is a glycoprotein-associated carbohydrate antigen discovered in 1979 that equals a sialylated hapten of the Lewis blood group antigen and is present in a wide variety of cells [8]. The CA 19-9 is not elevated only in pancreatic adenocarcinoma, but also in other neoplasia, especially in the digestive ones [7,9,10]. Despite substantial efforts made in recent years to identify more specific serum markers for pancreatic adenocarcinoma, to this point, CA 19-9 serves as the only validated serum biomarker. CA 19-9 was consistently and widely integrated into routine clinical practice for diagnostic and monitoring of pancreatic adenocarcinoma [7].

In this context, searching for new diagnosis serum markers remains an important field of research. We herein intended to test the utility of three serum cancer biomarkers in patients with pancreatic adenocarcinoma, namely the cytokeratin-19 fragments (CYFRA 21-1), osteopontin (OPN), and human epididymis protein 4 (HE4).

CYFRA 21-1 is an intermediate filament protein necessary for the cells’ stability, acting as a structural component of the cytoskeleton [2,8]. It is expressed in epithelial cells and released into circulation following tumor cell necrosis and apoptosis [8,10,11]. CYFRA 21-1 is recognized as a biomarker in several epithelial malignancies, like lung and breast cancers, and holds promise to be an independent tumoral marker for pancreatic adenocarcinoma [12,13]. As CYFRA 21-1 is a fragment of cytokeratin-19, a ductal epithelial marker upregulated during acinar-to-ductal metaplasia in early pancreatic carcinogenesis, its presence in serum may reflect the emergence of ductal phenotypes that characterize pancreatic cancer tumorigenesis [14,15,16].

OPN is a calcium-binding phosphorylated glycoprotein secreted by transformed cell lines and involved in multiple biological functions [17,18]. OPN presents a specific cell-binding motif, specifically the Arg-Gly-Asp (RGD) motif. The OPN arginine-glycine-aspartic acid (RGD) domain might interact with members of the integrin family [1,19]. By way of example, OPN can directly stimulate autophagy via integrin/CD44 and p38 mitogen-activated protein kinase (MAPK) signaling pathways. This multifunctional protein is highly expressed in various cancerous cells, being involved in cellular proliferation, survival, migration, invasion, and angiogenesis [13,20,21]. Therefore, OPN was proposed as potential valuable serum biomarker in several types of neoplasia, encompassing also the pancreatic adenocarcinoma [17,22].

HE4 is a secretory glycoprotein expressed in the epididymis, trachea, ovary, and endometrium [23]. HE4 is part of the whey-acidic-protein (WAP) family containing WAP-type four-disulfide core domains [23,24,25]. The exact physiological function of the WAP-related proteins is poorly understood, but it is supposed to work as extracellular protease inhibitors, involved in the regulation of extracellular matrix, cell migration, and cell invasion [24,25,26]. HE4 was initially identified as a serum tumor marker in ovarian and endometrial cancer, and has subsequently been reported in several non-gynecologic cancers, including pancreatic adenocarcinoma [23,27,28].

Therefore, the aim of this study was to assess the value of CYFRA 21-1, OPN, and HE4 in pancreatic adenocarcinoma, tumoral markers that are yet poorly characterized for this pathology.

## 2. Results

### 2.1. General Data

A total of 60 patients with confirmed pancreatic adenocarcinoma were included in this study. The male-to-female ratio was 1:1.3, and the mean age at inclusion was 68.4 ± 10.2 years. Also, serological samples from 14 patients without a known malignant disease were collected. Demographic, clinical, and laboratory baseline characteristics are summarized in Table 1 and Table S1 of Supplementary File.

Serum levels of CA 125, CA 19-9 or OPN were significantly higher in patients with pancreatic adenocarcinoma when compared to controls 61.4 (24.4; 145.7) versus 6.2 (3.0; 20.1) U/mL, *p* = 0.020; 907.9 (63.4; 11,273.2) U/mL versus 15.7 (4.1; 15.7) U/mL; *p* = 0.040 and 3.37 (1.84; 9.12) ng/mL versus 1.59 (1.09; 2.51) ng/mL; *p* = 0.003, but not for CYFRA 21-1 and HE4. However, in multivariate regression analysis, OPN was not an independent predictor of pancreatic cancer diagnosis (*p* > 0.05).

As expected, the serum cytolysis and cholestasis hepatic enzymes, as well as the white blood cells count, were higher in cases with pancreatic adenocarcinoma when compared to controls (see Supplementary File).

### 2.2. Cytokeratin 19-Fragments (CYFRA 21-1)

In bivariate analysis, the serum levels of CYFRA 21-1 correlated with those of OPN (r = 0.302; *p* = 0.009), HE4 (r = 0.485; *p* < 0.001), and CA 125 (r = 0.376; *p* = 0.037). In multivariate analysis adjusted for non-modifiable confounders, higher serum levels of OPN, HE4, and CA 125 were independently associated with increased CYFRA 21-1 in patients with pancreatic cancer: OR (95% CI) 4.190 (1.303–13.470); *p* = 0.016, 0.232 (0.071–0.761); *p* = 0.016, and 0.147 (0.027–0.801); *p* = 0.027, respectively.

Higher CYFRA 21-1 levels were more frequently noted in patients who experienced significant weight loss in the three months before the hospitalization (77.4% versus 57.1%; *p* = 0.021) (see Table 2 and Table S2 of Supplementary File).

Subsequently, in multivariate analysis, significant weight loss before admission was a predictor for higher CYFRA 21-1 serum levels: OR 95% (CI) 0.211 (0.048–0.922), *p* = 0.039.

### 2.3. Osteopontin (OPN)

Patients with pancreatic adenocarcinoma and high OPN levels also showed significantly higher serum levels of CYFRA 21-1 and HE4, but not of CA 125 or CA 19-9: 0.28 (0.19; 1.94) pg/mL versus 0.15 (0.11; 0.37) pg/mL; *p* = 0.006, 0.48 (0.22; 10.16) ng/mL versus 0.18 (0.10; 0.34) ng/mL; *p* = 0.000, 104.3 (48.3; 267.8) U/mL versus 33.5 (18.2; 119.3) U/mL; *p* = 0.090, and 1729.8 (75.4; 11,505.5) U/mL versus 550.0 (18.7; 11,195.7) U/mL; *p* = 0.988 respectively (see Table 3 and Table S3 of Supplementary File).

In multivariate analysis, adjusted for demographic variables, elevated serum levels of CYFRA 21-1 and HE4 were identified as predictors for higher OPN blood levels: OR 95% (CI) 0.249 (0.078–0.796); *p* = 0.019 and 0.245 (0.077–0.781); *p* = 0.017, respectively.

Further, patients with higher CYFRA 21-1 levels appear to have more severe background pathology in terms of significantly lower hemoglobin levels (11.8 (10.9; 12.5) versus 13.4 (12.2; 14.0) g/dL; *p* = 0.003) or lipase serum levels (88.0 (46.7;180.7) versus 21.5 (7.0; 35.7) U/L; *p* = 0.002), as well as weight loss in a higher proportion (24 (77.4%) versus 16 (57.1%); *p* = 0.021) (see Table 3 and Supplementary File).

### 2.4. Human Epididymis Protein 4 (HE4)

Patients with elevated HE4 levels exhibited significantly higher serum concentrations of CYFRA 21-1, OPN, CA 125, but also of CA 19-9: 0.37 (0.15; 1.94) pg/mL versus 0.17 (0.11; 0.28) pg/mL, *p* = 0.002; 6.60 (2.29; 13.64) ng/mL versus 2.24 (1.43; 3.99) ng/mL; *p* = 0.001, 137.6 (41.8; 442.3) U/mL versus 45.4 (17.3; 83.2) U/mL; *p* = 0.022 and 3610 (168.1; 31,335.2) U/mL versus 260.3 (15.4; 3298.1) U/mL; *p* = 0.041, respectively.

Patients with higher HE4 levels also had more frequent emesis as a symptom at presentation (see Table 4 and Table S4 of Supplementary File).

Increased levels of CYFRA 21-1 and OPN were predictors for higher HE4 serum levels: OR 95% (CI) 0.247 (0.076–0.801); *p* = 0.020 and 4.106 (1.297–13.00); *p* = 0.016, respectively.

### 2.5. Diagnostic Performance of Serum Biomarkers

Based on receiver operating characteristic (ROC) curve analysis, CA 19-9 demonstrated the highest diagnostic accuracy for pancreatic adenocarcinoma (AUC = 0.856, 95% CI 0.738–0.973, *p* = 0.043). In contrast, OPN, CYFRA 21-1, and HE4 showed substantially lower discriminatory performance, with AUC values ranging from 0.414 to 0.568, indicating that these biomarkers are markedly inferior to CA 19-9 for diagnostic purposes.

## 3. Discussion

The present study investigated the clinical relevance of three tumoral serum biomarkers—CYFRA 21-1, OPN, and HE4—in patients with pancreatic adenocarcinoma. There is a lack of information in the specialized literature regarding the appropriate cut-off values for many cancer biomarkers in general populations, as well as disease-specific subgroups. As no validated cut-offs are currently available for the biomarkers tested here in pancreatic adenocarcinoma, median values were used as cut-offs. Based on data from the healthy population, a cut-off of 1.8 ng/mL was initially settled for CYFRA 21-1 [2]. However, further research has shown that this cut-off is not sensitive enough to discriminate cancer [2]. In a different study, the cut-off of 0.2 pg/mL was useful for prognosis prediction in patients with hepatocellular carcinoma [29].

CYFRA 21-1 has been investigated as a diagnostic biomarker among several malignancies like cholangiocarcinoma (CCA) [30], malignant pleural effusion [31], lung cancer [12,32,33], head and neck cancer [12], gastrointestinal cancer, cervical cancer [12], bladder cancer [10], or urothelial carcinoma [10]. This biomarker has been studied not only as a diagnostic tool but also for its potential to predict prognosis or treatment response in cancers [8], including the non-small cell lung cancer [32,33], head and neck cancer [12], or the urothelial carcinoma [10].

In cholangiocarcinoma, the CYFRA 21–1 demonstrated higher specificity [8] and correlated more closely with the tumor stage than CA 19-9 [30]. Alike, the CYFRA 21-1 levels were associated with tumor size and local invasion in hepatocellular carcinoma in a different study [8]. In pancreatic cancer, CYFRA 21-1 has been shown to better discriminate locally advanced from metastatic cancer [6,9]. Unlike CA 19-9, CYFRA 21-1 blood levels are not influenced by the degree of jaundice [9].

In the present study, elevated CYFRA 21-1 levels were more frequently observed in patients with pancreatic adenocarcinoma who experienced significant weight loss or lipase elevation. Additionally, the levels of all the other four markers tested (CA 125, CA 19-9, OPN, and HE4) were significantly higher in patients with raised CYFRA 21-1 concentrations.

The name “osteopontin”, through its two elements “osteo” means bone, and “pontin” means bridge, expresses the function of this molecule. OPN is an important element of the bone matrix and plays fundamental roles in bone formation, remodeling, and resorption [19,21].

OPN has been reported to be expressed at higher levels in tumor cells compared with normal tissues across various epithelial malignancies, including breast, lung, liver, stomach, and ovarian [17]. Of the five OPN isoforms [21], characterized according to the distinct splice of exons 4 and 5 [22], OPN-a and OPN-c are highly expressed in breast cancer, OPN-b in gliomas, and OPN-d and OPN-e in esophageal adenocarcinoma. Also, OPN-a and OPN-b were revealed to be expressed in lung cancer [22,34].

Furthermore, OPN levels were found to be correlated with the tumoral stage in breast, lung, gastric, ovarian, and melanoma cancers [17,18]. The OPN-c isoform may promote invasion and metastasis through activation of the JAK/STAT3 signaling pathway [22].

Additionally, OPN has also been proposed as a novel immune checkpoint [21] and as a potential anti-cancer target for various neoplasms [35] that could potentially complement the classical anti-PD-1 immunotherapy [1]. In vitro models have shown that OPN mRNA is upregulated in pancreatic cancer cell lines [35]. Moreover, smoking, a known risk factor for pancreatic cancer, has been shown to induce OPN expression through nicotine exposure [22,35].

On the other hand, the high mortality rate of pancreatic cancer is related to metastasis from the early stages. MMP-9 and VEGF are two important players in tumoral progression and metastasis, with their expression upregulated by OPN [35]. Inhibition of OPN signaling has therefore been suggested as a potential strategy to limit liver metastasis in pancreatic cancer [1].

Regarding the role of OPN in pancreatic inflammation under benign conditions, such as chronic pancreatitis, this glycoprotein may exacerbate local inflammatory responses [3,22]. Therefore, it is thought to play a role in the activation of pancreatic stellate cells and fibrotic remodeling by recruitment of inflammatory cells and further cytokine production [22]. In this perspective, as the OPN expression could be upregulated in chronic pancreatitis, the reliability of OPN as a tumoral marker in pancreatic neoplasia was questioned [21].

The results presented in the present work suggest that patients with pancreatic cancer have higher levels of OPN when compared to healthy individuals. Notably, the OPN serum levels were not correlated with CA 19-9, showing a distinct pattern. This observation supports the potential role of OPN in capturing aspects of pancreatic cancer not adequately represented by conventional tumor markers. Importantly, these findings should be interpreted in a complementary context, as none of the biomarkers investigated here could replace CA 19-9, but rather provide additional biological and clinical information.

Similarly to other cancer biomarkers, increased HE4 expression has been reported in gynecologic and pulmonary malignancies [28,36]. Moreover, in pancreatic cancer, HE4 expression is higher in tumor tissues compared with normal and adjacent non-tumorous pancreatic tissues [36]. The HE4 upregulation in upper gastrointestinal neoplasia begins as early as the dysplastic preneoplastic stages of pancreatic carcinogenesis [37]. In addition, the HE4 increased tissue distribution was linked to more aggressive tumoral phenotypes [24,38]. Elevated serum HE4 levels have also been found in patients with pancreatic adenocarcinoma compared to controls [24,25,36,39], even in the early stages [40].

Serum HE4 was labeled in 2009 by the US Food and Drug Administration (FDA) as a tumoral marker in ovarian cancer for both diagnosis and monitoring [26]. Further, in 2011, the algorithm Risk of Ovarian Malignancy Algorithm (ROMA), combining HE4 with CA 125, was introduced for the evaluation of female patients with a pelvic mass [26]. Similarly, combining HE4 with CA 19-9 for better diagnostic sensitivity in pancreatic adenocarcinoma was discussed [24,36]. We identified higher CA 19-9 and OPN levels in patients with increased HE4 serum levels.

HE4 has also been linked to reduced sensitivity to paclitaxel, the first-line chemotherapy drug used for pancreatic cancer and other malignancies [23,41]. The neoplastic tissues with over-expressed HE4 present a decreased sensitivity to paclitaxel [23] and can induce chemoresistance [42]. In this context, a potential role as a therapeutic target for pancreatic cancer for HE4 was suggested [42,43].

This research has several limitations. First, the number of patients included is not large enough to allow a statistically meaningful sample size. Secondly, this research was conducted in a single center, and, given this setting, a selection bias could have occurred. Further, a notable limitation of this study is the age imbalance between the case and control groups, which should be considered when interpreting the results. Additionally, median-based cut-offs were applied for exploratory purposes, given that no validated thresholds are available for pancreatic cancer.

However, there are also important strengths of the current research, such as the prospective design. Also, three important serum biomarkers, CYFRA 21-1, OPN, and HE4 were simultaneously determined. Notably, this research novelty lies in proving new data over three tumoral biomarkers which have been poorly studied in patients with pancreatic cancer and whose significance remains not understood in this subset of patients.

Future research should evaluate whether dynamic changes in CYFRA 21-1, OPN, and HE4 after oncological therapy can serve as reliable indicators for monitoring therapeutic response and identifying recurrence, thereby establishing whether these markers should be regarded as complementary to, and not a substitute for, the currently CA 19-9-based approach.

## 4. Materials and Methods

### 4.1. Study Population

We herein present a single-center cross-sectional study with prospective inclusion of adult patients (more than 18 years) diagnosed with pancreatic adenocarcinoma admitted into the Gastroenterology Department of the Military Central University Hospital. All cases with a confirmed diagnosis of pancreatic adenocarcinoma were prospectively and consecutively enrolled at the time of hospital admission. The diagnosis of adenocarcinoma was sustained in all cases by histopathological exam.

Overall, 60 consecutive patients diagnosed with pancreatic adenocarcinoma and 14 controls without any known neoplastic disease were included in the study, between 1 February 2023 and 1 November 2023.

Standard study forms were completed in all cases at inclusion. Clinical data and medical records were similarly analyzed and registered. Background variables concerning general data, symptomatology, laboratory tests, and paraclinical data at admission were gathered in all cases. For all study participants, the same clinical and biological data were acquired to ensure group homogeneity.

### 4.2. Sample Collection

Blood samples were collected at inclusion from all patients, further centrifuged for 15 min at 4000 rpm, and then stored in a −70 °C ultra-low temperature freezer. After controlled thawing, the samples were processed together in a single step for all patients, with assessments conducted for the three markers CYFRA 21-1, OPN, and HE4.

Antibody-immobilized beads, quality controls (2), serum matrix, and standards (7) were prepared according to the producer’s recommendations. Serum samples were diluted 1:6 with a serum matrix. First, 200 μL of assay buffer was added to all the plate wells, and the sealed plate was kept for 10 min on a shaker to remove all the residual amounts. A total of 25 μL of assay buffer was added to background wells, 25 μL of standards and controls, and 25 μL of appropriate matrix to the Background, Standard, and Control wells. In the sample wells, we introduced 25 μL of assay buffer and then 25 μL of diluted samples. A total of 25 μL of mixed beads was added to each well, and the plate was incubated at 4 °C overnight for 16–18 h on the shaker. The content from all the wells was removed and washed 3 times, and we added 25 μL of detection antibodies, followed by incubation for 1 h on the plate shaker at room temperature. Then, 25 μL of streptavidin-phycoerythrin was added to each well, followed by incubation on the plate shaker for 30 min. In the end, 100 μL of sheath fluid was added, and we read the mean fluorescence intensity. Finally, the concentration for each sample was calculated.

The analysis was performed using a multianalyte panel from the selected Human Circulating Cancer Biomarker Panel (the EMD Millipore Corporation, Billerica, MA, USA, Cat. No. HCCBP1MAG-58K was used in this study). Seven working standards were generated by serial dilution of the reconstituted standard provided in the kit. Two quality control samples were included in each plate run.

The Luminex^®^ system was employed for optimal measurement and detection of the biomarkers. Milliplex Analysts were used for data analysis. The laboratory kit minimum detection limit for CYFRA 21-1 is 0.2 pg/mL, for OPN is 0.3 ng/mL, and for HE4 is 0.1 ng/mL.

Oral and written informed consents were obtained from all study participants, cases, and controls. The local Ethics Committee approved the study protocol for the research presented here.

### 4.3. Statistical Analysis

Categorical values are presented as mean ± standard deviation (SD) or as the median (med) with an interquartile range (IQR), where q25 represents the first quartile and q75 the third quartile, depending on their distribution. Nominal variables are expressed as percentages.

For bivariate analysis, the chi-square test was applied to assess differences in categorical variables, while the Mann–Whitney U test was used for variables that were not normally distributed. The non-parametric Spearman test (r = Spearman’s rho coefficient) was used for bivariate correlations. The median value was set as a cut-off in the absence of a formal agreement or established standard.

A multivariate analysis by binary logistic regression was performed to test the variables as possible predictors for the outcome considered. The results were expressed as odds ratios (ORs) with 95% confidence intervals (CIs). The *p* probability cut-off for rejecting the null hypothesis was 0.05. The statistical analyses were performed using the IBM SPSS Statistics, version 25 (IBM Corp., Armonk, NY, USA).

## 5. Conclusions

In conclusion, we herein present new data regarding three important cancer biomarkers—CYFRA 21-1, OPN, and HE4—in patients with pancreatic cancer. Even if the serum blood levels of the cancer biomarkers tested showed intercorrelated serum levels in patients with pancreatic adenocarcinoma, they did not demonstrate diagnostic accuracy and therefore cannot be considered useful markers for pancreatic cancer diagnosis, especially when compared with the established performance of CA 19-9. Nevertheless, the observed variations of these biomarkers likely reflect secondary cancer-associated phenomena like inflammation, cholestasis, or cancer-associated weight loss, supporting new research directions focused on the use of these biomarkers in providing adjunctive information.

## Figures and Tables

**Table 1:** General characteristics for control and patients with pancreatic adenocarcinoma included in the study.

Parameter	Controls n1 = 14	Cases n2 = 60	*p*-Value
Gender, M/F (%M)	8/6 (57.1%)	26/34 (43.3%)	0.351
Age years, med (q1; q3)	51.0 (46.0; 65.0)	69.5 (64.2; 76.0)	0.001
Leucocytes/μL, med (q1; q3)	6530 (5090; 8672)	8100 (6675; 10,975)	0.045
Neutrophils/μL, med (q1; q3)	3990 (2615; 5437)	5570 (4425; 8040)	0.005
Hemoglobin g/dL, med (q1; q3)	13.2 (11.6; 14.7)	12.4 (11.3; 13.7)	0.172
Thrombocytes/μL, med (q1; q3)	245.5 (151.2; 324.0)	280.0 (205.5; 344.5)	<0.001
ESR mm/h, med (q1; q3)	19.0 (14.5; 45.5)	31.0 (20.7; 63.7)	0.119
CRP mg/dL, med (q1; q3)	3.6 (1.1; 16.7)	14.5 (4.9; 86.0)	0.022
Bilirubin mg/dL, med (q1; q3)	0.7 (0.4; 1.3)	1.4 (0.5; 7.4)	0.062
Creatinine mg/dL, med (q1; q3)	0.7 (0.6; 0.8)	0.7 (0.6; 0.9)	0.724
ALAT U/L, med (q1; q3)	21.0 (18.5; 29.5)	45.0 (26.0; 140.0)	0.002
GGT U/L, med (q1; q3)	29.0 (18.0; 47.0)	308.5 (38.5; 996.5)	<0.001
ALP U/L, med (q1; q3)	68.0 (66.0; 107.0)	197.0 (82.5; 492.5)	0.007
Amylase U/L, med (q1; q3)	69.0 (60.0; 77.5)	60.0 (36.0; 93.5)	0.543
Lipase U/L, med (q1; q3)	8.0 (8.0; 8.0)	40.5 (18.5; 105.5)	0.222
CA 125 U/mL, med (q1; q3)	6.2 (3.0; 20.1)	61.4 (24.4; 145.7)	0.020
CA 19-9 U/mL, med (q1; q3)	15.7 (4.1; 15.7)	907.9 (63.4; 11,273.2)	0.040
CYFRA 21-1 pg/mL, med (q1; q3)	0.23 (0.21; 0.33)	0.24 (0.13; 0.42)	0.866
OPN ng/mL, med (q1; q3)	1.59 (1.09; 2.51)	3.37 (1.84; 9.12)	0.003
HE4 ng/mL, med (q1; q3)	0.25 (0.09; 4.63)	0.29 (0.15; 0.69)	0.304

Abbreviations: ALAT—alanine aminotransferase; ALP—alkaline phosphatase; CA—cancer antigen; CRP—C reactive protein; CYFRA 21-1—Cytokeratin 19-fragments; ESR—erythrocyte sedimentation rate; GGT—gamma-glutamyl transferase; OPN—osteopontin; HE4—human epididymis protein 4. *p*-value < 0.05 was considered statistically significant.

**Table 2:** Results of the analysis centered on the CYFRA 21-1 serum level.

Parameter	Low CYFRA 21-1	High CYFRA 21-1	*p*-Value
Gender, M/F (%M)	12/16 (42.9%)	14/17 (45.2%)	0.859
Age, years med (q1; q3)	68.5 (61.7; 74.7)	70.0 (65.0; 77.0)	0.323
BMI, kg/m^2^ med (q1; q3)	25.9 (22.6; 29.2)	23.8 (21.8; 27.5)	0.062
Weight loss, n (%)	16 (57.1%)	24 (77.4%)	0.021
Leucocytes/μL, med (q1; q3)	7890 (6340; 9660)	8890 (6675; 11,435)	0.380
Neutrophils/μL, med (q1; q3)	4990 (4230; 6420)	6520 (5070; 9090)	0.027
Hemoglobin g/dL, med (q1; q3)	13.4 (12.2; 14.0)	11.8 (10.9; 12.5)	0.003
Thrombocytes/μL, med (q1; q3)	276 (108; 322)	290 (198; 344.5)	0.850
ALAT U/L, med (q1; q3)	66.5 (29.0; 327.0)	50.0 (20.0; 162.0)	0.240
GGT U/L, med (q1; q3)	601.0 (49.0; 1042.0)	199.0 (35.0; 715.0)	0.250
Amylase U/L, med (q1; q3)	62.0 (46.5; 86.5)	60.0 (34.0; 101.0)	0.412
Lipase U/L, med (q1; q3)	21.5 (7.0; 35.7)	88.0 (46.7;180.7)	0.002
CA 125 U/mL, med (q1; q3)	32.9 (17.3; 95.5)	93.6 (49.6; 412.5)	0.014
CA 19-9 U/mL, med (q1; q3)	180.3 (17.6; 1847.1)	3820.8 (158.3; 29,953.0)	0.027
OPN, ng/mL med (q1; q3)	2.4 (1.4; 4.0)	6.1 (2.1; 11.8)	0.006
HE4, ng/mL med (q1; q3)	1.85 (1.04; 3.56)	5.46 (1.90; 10.65)	<0.001

Abbreviations: ALAT—alanine aminotransferase; CA—cancer antigen; CYFRA 21-1—Cytokeratin 19-fragments; GGT—gamma-glutamyl transferase; OPN—osteopontin; HE4—human epididymis protein 4. *p*-value < 0.05 was considered statistically significant.

**Table 3:** Results of the analysis centered on the osteopontin serum level.

Parameter	Low OPN	High OPN	*p*-Value
Gender, M/F (%M)	10/20 (33.3%)	16/14 (53.3%)	0.118
Age, years med (q1; q3)	68.5 (55.7; 75.0)	70.0 (67.7; 77.0)	0.082
BMI, kg/m^2^ med (q1; q3)	24.8 (21.8; 28.0)	24.6 (21.9; 28.0)	0.678
Leucocytes/μL, med (q1; q3)	7810 (6300; 9570)	9265 (6792; 11,780)	0.162
Neutrophils/μL, med (q1; q3)	5360 (4230; 6890)	6095 (4587; 8812)	0.164
Hemoglobin g/dL, med (q1; q3)	12.7 (11.8; 13.9)	12.1 (10.8; 13.6)	0.243
Thrombocytes/μL, med (q1; q3)	280.0 (208.0; 347.0)	271.5 (191.2; 345.2)	0.898
ALAT U/L, med (q1; q3)	37.0 (23.7; 162.0)	50.0 (19.0; 162.0)	0.335
GGT U/L, med (q1; q3)	601.0 (43.0; 1067.0)	252.0 (36.0; 648.5)	0.327
Amylase U/L, med (q1; q3)	61.0 (39.0; 82.7)	60.0 (35.0; 101.5)	0.795
Lipase U/L, med (q1; q3)	59.5 (23.2; 240.2)	31.5 (10.2; 105.5)	0.316
CA 125 U/mL, med (q1; q3)	33.5 (18.2; 119.3)	104.3 (48.3; 267.8)	0.090
CA 19-9 U/mL, med (q1; q3)	550.0 (18.7; 11,195.7)	1729.8 (75.4; 11,505.5)	0.988
CYFRA 21-1, pg/mL med (q1; q3)	0.15 (0.11; 0.37)	0.28 (0.19; 1.94)	0.006
HE4, ng/mL med (q1; q3)	0.18 (0.10; 0.34)	0.48 (0.22; 10.16)	<0.001

Abbreviations: ALAT—alanine aminotransferase; CA—cancer antigen; CYFRA 21-1—Cytokeratin 19-fragments; GGT—gamma-glutamyl transferase; OPN—osteopontin; HE4—human epididymis protein 4. *p*-value < 0.05 was considered statistically significant.

**Table 4:** Results of the analysis centered on the HE4 serum level.

Parameter	Low HE4	High HE4	*p*-Value
Gender, M/F (%M)	10/20 (33.3%)	16 (53.3%)	0.118
Age, years med (q1; q3)	68.0 (55.7; 72.5)	73.5 (65.7; 77.0)	0.030
BMI, kg/m^2^ med (q1; q3)	25.9 (21.9; 29.2)	24.2 (21.8; 27.1)	0.283
Smoke, n (%)	6 (20.0%)	3 (10.0%)	0.302
Emesis, n (%)	6 (20.0%)	13 (43.3%)	0.046
Leucocytes/μL, med (q1; q3)	7025 (5535; 9527)	9940 (7330; 11,965)	0.009
Neutrophils/μL, med (q1; q3)	4960 (4247; 6372)	6780 (5150; 9165)	0.029
Hemoglobin g/dL, med (q1; q3)	12.9 (11.9; 13.8)	11.8 (10.1; 13.2)	0.027
Thrombocytes/μL, med (q1; q3)	270.5 (201.5; 320.7)	294.0 (237.0; 372.0)	0.158
ALAT U/L, med (q1; q3)	59.0 (20.0; 364.5)	50.0 (21.0; 125.0)	0.399
GGT U/L, med (q1; q3)	395.0 (33.0; 1067.0)	293.0 (52.5; 669.0)	0.657
Amylase U/L, med (q1; q3)	60.0 (37.0; 110.0)	60.0 (35.7; 93.2)	0.521
Lipase U/L, med (q1; q3)	67.0 (20.0; 167.0)	31.5 (15.5; 97.5)	0.396
CA 125 U/mL, med (q1; q3)	45.4 (17.3; 83.2)	137.6 (41.8; 442.3)	0.022
CA 19-9 U/mL, med (q1; q3)	260.3 (15.4; 3298.1)	3610.0 (168.1; 31,335.2)	0.041
CYFRA 21-1, pg/mL med (q1; q3)	0.17 (0.11; 0.28)	0.37 (0.15; 1.94)	0.002
OPN, ng/mL med (q1; q3)	2.24 (1.43; 3.99)	6.60 (2.29; 13.64)	0.001
HE4, ng/mL med (q1; q3)	0.15 (0.10; 0.19)	0.65 (0.37; 10.80)	<0.001

Abbreviations: ALAT—alanine aminotransferase; CA—cancer antigen; CYFRA 21-1—Cytokeratin 19-fragments; GGT—gamma-glutamyl transferase; OPN—osteopontin; HE4—human epididymis protein 4. *p*-value < 0.05 was considered statistically significant.

## Data Availability

The original contributions presented in this study are included in the article/Supplementary Materials. Further inquiries can be directed to the corresponding authors.

## Supplementary Materials

The following supporting information can be downloaded at: https://www.mdpi.com/article/10.3390/ijms27031562/s1.

## Abbreviations

The following abbreviations are used in this manuscript:

ALAT	Alanine aminotransferase
ALP	Alkaline phosphatase
ASAT	Aspartate aminotransferase
CA	Cancer antigen
CYFRA 21-1	Cytokeratin 19-fragments
CRP	C-reactive protein
ESR	Erythrocyte sedimentation rate
HDL	High-density lipoprotein
GGT	Gamma-glutamyl transferase
OPN	Osteopontin
HE4	Human epididymis protein 4
