# Supplementary material for: Clinical Significance of Cytokeratin 19-Fragments (CYFRA 21-1), Osteopontin (OPN) and Human Epididymis Protein 4 (HE4) in Pancreatic Adenocarcinoma
Source: Int J Mol Sci. 2026 Feb 5;27(3):1562. doi: 10.3390/ijms27031562 (PMC12898393; doi:10.3390/ijms27031562)
Supplement: Supplementary file 1 [file ijms-27-01562-s001.zip › ijms-4109823-supplementary.pdf]

Table S1. General characteristics for control and patients with pancreatic adenocarcinoma included in the study (extended data compared to those presented in the article).

**Parameter**	**Controls** n1=14	**Cases** n2=60	**p-value**
Gender, M/F (%M)	8/ 6 (57.1%)	26/ 34 (43.3%)	0.351
Age, years med (q1; q3)	51.0 (46.0; 65.0)	69.5 (64.2; 76.0)	0.001
BMI, kg/m^2 med (q1; q3)	-	24.7 (21.9; 28.0)	-
Smoke, n (%)	-	9 (15.0%)	-
Alcohol, n (%)	-	15 (25.0%)	-
Meat-rich diet, n (%)	-	20 (33.3%)	-
Sweet-rich diet, n (%)	-	16 (26.7%)	-
High-fat diet, n (%)	-	13 (21.7%)	-
Abdominal pain, n (%)	-	33 (55.0%)	--
Heartburn, n (%)	-	18 (30.0%)	-
Vomiting, n (%)	-	19 (31.7%)	-
Nausea, n (%)	-	22 (36.7%)	-
Jaundice, n (%)	-	18 (30.0%)	-
Weight loss, n (%)	-	41 (68,3%)	-
CYFRA21-1, pg/mL med (q1; q3)	0.23 (0.21; 0.33)	0.24 (0.13; 0.42)	0.866
CYFRA21-1 cut-off 0.24 pg/mL	7 (50.0%)	30 (50.0%)	0.864
OPN, ng/mL med (q1; q3)	1.59 (1.09; 2.51)	3.37 (1.84; 9.12)	0.003
OPN cut-off 3.3 ng/mL	1 (7.1%)	31 (51.7%)	0.003
HE4, ng/mL med (q1; q3)	0.25 (0.09; 4.63)	0.29 (0.15; 0.69)	0.304
HE4 cut-off ng/mL	5 (35.7%)	30 (50.0%)	0.335
Leucocytes/ µL, med (q1; q3)	6530 (5090; 8672)	8100 (6675; 10975)	0.045
Neutrophils/ µL, med (q1; q3)	3990 (2615; 5437)	5570 (4425; 8040)	0.005
Lymphocytes/ µL, med (q1; q3)	1920 (1122; 2262)	1610 (1145; 2137)	0.370
Hemoglobin g/dL, med (q1; q3)	13.2 (11.6; 14.7)	12.4 (11.3; 13.7)	0.172
Thrombocytes/ µL, med (q1; q3)	245.5 (151.2; 324.0)	280.0 (205.5; 344.5)	0.000
ESR mm/, med (q1; q3)	19.0 (14.5; 45.5)	31.0 (20.7; 63.7)	0.119
CRP mg/dL, med (q1; q3)	3.6 (1.1; 16.7)	14.5 (4.9; 86.0)	0.022
Bilirubin, mg/dL med (q1; q3)	0.7 (0.4; 1.3)	1.4 (0.5; 7.4)	0.062
Creatinine, mg/dL med (q1; q3)	0.7 (0.6; 0.8)	0.7 (0.6; 0.9)	0.724
Uric acid, mg/dL med (q1; q3)	4.9 (4.3; 6.7)	4.4 (3.7; 5.7)	0.283
ASAT U/L, med (q1; q3)	20.0 (14.0; 26.5)	56.5 (20.0; 186.5)	0.002
ALAT U/L, med (q1; q3)	21.0 (18.5; 29.5)	45.0 (26.0; 140.0)	0.002
GGT U/L, med (q1; q3)	29.0 (18.0; 47.0)	308.5 (38.5; 996.5)	0.000
ALP U/L, med (q1; q3)	78.0 (66.0; 107.0)	197.0 (82.5; 492.5)	0.007
Amylase U/L, med (q1; q3)	69.0 (60.0; 77.5)	60.0 (36.0; 93.5)	0.543
Lipase U/L, med (q1; q3)	8.0 (8.0; 8.0)	40.5 (18.5; 105.5)	0.222
Total cholesterol mg/dL, med (q1; q3)	198.0 (156.0; 248.0)	191.0 (122.7; 214.2)	0.470
HDL-cholesterol mg/dL, med (q1; q3)	61.4 (47.4; 79.1)	44.2 (33.1; 50.2)	0.025
Triglycerides mg/dL, med (q1; q3)	81.0 (70.0; 137.0)	112.0 (94.0; 223.0)	0.110
Albumin g/dL, med (q1; q3)	4.1 (3.5; 4.5)	3.9 (3.5; 4.2)	0.322
CA 19-9 U/ mL, med (q1; q3)	15.7 (4.1; 15.7)	907.9 (63.4; 11273.2)	0.040
CA 125 U/ mL, med (q1; q3)	6.2 (3.0; 20.1)	61.4 (24.4; 145.7)	0.020

Abbreviations*: ALAT - alanine aminotransferase; ALP - alkaline phosphatase; ASAT - aspartate aminotransferase; CA - cancer antigen; CYFRA 21-1 - Cytokeratin 19-fragments; CRP – C-reactive protein; ESR – erythrocyte sedimentation rate; HDL - high-density lipoprotein; GGT - gamma-glutamyl transferase; OPN - osteopontin; HE4 - human epididymis protein 4.*
p-value <0.05 was considered statistically significant.

Table S2. Comparative analysis of the parameters analyzed in the patients with pancreatic cancer included in study in relation to low versus respectively high CYFRA-21 levels in relation to the proposed cut-off (extended data compared to those presented in the article).

**Parameter**	**Cases** n=60	**Low CYFRA21-1** < 0.24pg/mL n1=28	**High CYFRA21-1** >0.24pg/mL n1=31	**p-value**
Gender, M/F (%M)	26/ 34 (43.3%)	12/16 (42.9%)	14/17 (45.2%)	0.859
Age, years med (q1; q3)	69.5 (64.2; 76.0)	68.5 (61.7; 74.7)	70.0 (65.0; 77.0)	0.323
BMI, kg/m^2 med (q1; q3)	24.7 (21.9; 28.0)	25.9 (22.6; 29.2)	23.8 (21.8; 27.5)	0.062
Smoke, n (%)	9 (15.0%)	7 (25.0%)	2 (6.5%)	0.061
Alcohol, n (%)	15 (25.0%)	9 (32.1%)	6 (19.4%)	0.326
Meat-rich diet, n (%)	20 (33.3%)	14 (50.0%)	6 (19.4%)	0.026
Sweet-rich diet, n (%)	16 (26.7%)	9 (32.1%)	7 (22.6%)	0.501
High-fat diet, n (%)	13 (21.7%)	6 (21.4%)	7 (22.6%)	0.807
Abdominal pain, n (%)	33 (55.0%)	17 (60.7%)	16 (51.6%)	0.646
Heartburn, n (%)	18 (30.0%)	9 (32.1%)	9 (29.0%)	0.767
Vomiting, n (%)	19 (31.7%)	11 (39.3%)	8 (25.8%)	0.291
Nausea, n (%)	22 (36.7%)	10 (35.7%)	12 (38.7%)	0.575
Jaundice, n (%)	18 (30.0%)	10 (35.7%)	8 (25.8%)	0.497
Weight loss, n (%)	41 (68.3%)	16 (57.1%)	24 (77.4%)	0.021
CYFRA21-1, pg/mL med (q1; q3)	0.24 (0.13; 0.42)	0.12 (0.09; 0.15)	0.41 (0.29; 1.38)	<0.001
OPN, ng/mL med (q1; q3)	3.37 (1.84; 9.12)	2.4 (1.4; 4.0)	6.1 (2.1; 11.8)	0.006
HE4, ng/mL med (q1; q3)	0.29 (0.15; 0.69)	1.85 (1.04; 3.56)	5.46 (1.90; 10.65)	0.000
Leucocytes/ µL, med (q1; q3)	8100 (6675; 10975)	7890 (6340; 9660)	8890 (6675; 11435)	0.380
Neutrophils/ µL, med (q1; q3)	5570 (4425; 8040)	4990 (4230; 6420)	6520 (5070; 9090)	0.027
Lymphocytes/ µL, med (q1; q3)	1610 (1145; 2137)	1740 (1330; 2117)	1320 (875; 2125)	0.066
Hemoglobin g/dL, med (q1; q3)	12.4 (11.3; 13.7)	13.4 (12.2; 14.0)	11.8 (10.9; 12.5)	0.003
Thrombocytes/ µL, med (q1; q3)	280.0 (205.5; 344.5)	276 (108; 322)	290 (198; 344.5)	0.850
ESR mm/, med (q1; q3)	31.0 (20.7; 63.7)	44.0 (19.0; 66.0)	30.0 (21.5; 62.0)	0.908
CRP mg/dL, med (q1; q3)	14.5 (4.9; 86.0)	8.3 (2.9; 48.6)	23.7 (8.4; 126.6)	0.239
Bilirubin, mg/dL med (q1; q3)	1.4 (0.5; 7.4)	2.3 (0.5; 7.8)	1.2 (0.5; 9.4)	0.491
Creatinine, mg/dL med (q1; q3)	0.7 (0.6; 0.9)	0.7 (0.5; 0.7)	0.8 (0.6; 1.0)	0.029
Uric acid, mg/dL med (q1; q3)	4.4 (3.7; 5.7)	4.4 (3.6; 5.1)	4.3 (3.7; 5.9)	0.626
ASAT U/L, med (q1; q3)	56.5 (20.0; 186.5)	62.0 (27.0; 144.5)	50.0 (24.0; 140.0)	0.671
ALAT U/L, med (q1; q3)	45.0 (26.0; 140.0)	66.5 (29.0; 327.0)	50.0 (20.0; 162.0)	0.240
GGT U/L, med (q1; q3)	308.5 (38.5; 996.5)	601.0 (49.0; 1042.0)	199.0 (35.0; 715.0)	0.250
ALP U/L, med (q1; q3)	197.0 (82.5; 492.5)	168.0 (85.0; 408.0)	220.0 (68.7; 586.2)	0.937
Amylase U/L, med (q1; q3)	60.0 (36.0; 93.5)	62.0 (46.5; 86.5)	60.0 (34.0; 101.0)	0.412
Lipase U/L, med (q1; q3)	40.5 (18.5; 105.5)	88.0 (46.7;180.7)	21.5 (7.0; 35.7)	0.002
Total cholesterol mg/dL, med (q1; q3)	191.0 (122.7; 214.2)	196.5 (131.5; 234.7)	190.0 (120.0; 209.0)	0.844
HDL-cholesterol mg/dL, med (q1; q3)	44.2 (33.1; 50.2)	43.1 (32.6; 49.0)	44.3 (32.5; 67.6)	0.673
Triglycerides mg/dL, med (q1; q3)	112.0 (94.0; 223.0)	121.0 (94.0; 286.0)	98.0 (83.2; 188.2)	0.329
Albumin g/dL, med (q1; q3)	3.9 (3.5; 4.2)	4.1 (3.7; 4.2)	3.9 (3.2; 4.2)	0.144
CA 19-9 U/ mL, med (q1; q3)	907.9 (63.4; 11273.2)	180.3 (17.6; 1847.1)	3820.8 (158.3; 29953.0)	0.027
CA 125 U/ mL, med (q1; q3)	61.4 (24.4; 145.7)	32.9 (17.3; 95.5)	93.6 (49.6; 412.5)	0.014

Abbreviations*: ALAT - alanine aminotransferase; ALP - alkaline phosphatase; ASAT - aspartate aminotransferase; CA - cancer antigen; CYFRA 21-1 - Cytokeratin 19-fragments; CRP – C-reactive protein; ESR – erythrocyte sedimentation rate; HDL - high-density lipoprotein; GGT - gamma-glutamyl transferase; OPN - osteopontin; HE4 - human epididymis protein 4.*
p-value <0.05 was considered statistically significant.

Table S3. Comparative analysis of the parameters analyzed in the patients with pancreatic cancer included in study in relation to low versus respectively high OPN levels in relation to the proposed cut-off (extended data compared to those presented in the article).

**Parameter**	**Cases**<br>n=60	**Low OPN**<br>< 3.3ng/mL<br>n1=30	**High OPN**<br>> 3.3ng/mL<br>n2=30	**p-value**
Gender, M/F (%M)	26/ 34 (43.3%)	10/20 (33.3%)	16/14 (53.3%)	0.118
Age, years med (q1; q3)	69.5 (64.2; 76.0)	68.5 (55.7; 75.0)	70.0 (67.7; 77.0)	0.082
BMI, kg/m^2 med (q1; q3)	24.7 (21.9; 28.0)	24.8 (21.8; 28.0)	24.6 (21.9; 28.0)	0.678
Smoke, n (%)	9 (15.0%)	6 (20.0%)	3 (10.0%)	0.358
Alcohol, n (%)	15 (25.0%)	8 (26.7%)	7 (23.3%)	0.949
Meat-rich diet, n (%)	20 (33.3%)	14 (46.7%)	6 (20.0%)	0.066
Sweet-rich diet, n (%)	16 (26.7%)	7 (23.3%)	9 (30.0%)	0.402
High-fat diet, n (%)	13 (21.7%)	6 (20.0%)	9 (30.0%)	0.594
Abdominal pain, n (%)	33 (55.0%)	17 (56.7%)	7 (23.3%)	0.646
Heartburn, n (%)	18 (30.0%)	7 (23.3%)	16 (53.3%)	0.288
Vomiting, n (%)	19 (31.7%)	7 (23.3%)	11 (36.7%)	0.154
Nausea, n (%)	22 (36.7%)	10 (33.3%)	12 (40.0%)	0.424
Jaundice, n (%)	18 (30.0%)	6 (20.0%)	12 (40.0%)	0.066
Weight loss, n (%)	41 (68,3%)	18 (60.0%)	23 (76.7%)	0.267
CYFRA21-1, pg/mL med (q1; q3)	0.24 (0.13; 0.42)	0.15 (0.11; 0.37)	0.28 (0.19; 1.94)	0.006
OPN, ng/mL med (q1; q3)	3.37 (1.84; 9.12)	1.84 (1.40; 2.31)	9.0 (5.1; 14.7)	<0.001
HE4, ng/mL med (q1; q3)	0.29 (0.15; 0.69)	0.18 (0.10; 0.34)	0.48 (0.22; 10.16)	<0.001
Leucocytes/ µL, med (q1; q3)	8100 (6675; 10975)	7810 (6300; 9570)	9265 (6792; 11780)	0.162
Neutrophils/ µL, med (q1; q3)	5570 (4425; 8040)	5360 (4230; 6890)	6095 (4587; 8812)	0.164
Lymphocytes/ µL, med (q1; q3)	1610 (1145; 2137)	1720 (1300; 2150)	1420 (910; 2125)	0.244
Hemoglobin g/dL, med (q1; q3)	12.4 (11.3; 13.7)	12.7 (11.8; 13.9)	12.1 (10.8; 13.6)	0.243
Thrombocytes/ µL, med (q1; q3)	280.0 (205.5; 344.5)	280.0 (208.0; 347.0)	271.5 (191.2; 345.2)	0.898
ESR mm/, med (q1; q3)	31.0 (20.7; 63.7)	28.0 (19.2; 63.7)	31.0 (26.2; 64.5)	0.419
CRP mg/dL, med (q1; q3)	14.5 (4.9; 86.0)	9.9 (2.3; 39.2)	17.4 (5.5; 132.7)	0.100
Bilirubin, mg/dL med (q1; q3)	1.4 (0.5; 7.4)	0.8 (0.5; 5.5)	3.3 (0.6; 11.1)	0.173
Creatinine, mg/dL med (q1; q3)	0.7 (0.6; 0.9)	0.7 (0.5; 0.8)	0.7 (0.6; 1.0)	0.269
Uric acid, mg/dL med (q1; q3)	4.4 (3.7; 5.7)	4.0 (3.5; 6.3)	4.7 (3.9; 5.6)	0.425
ASAT U/L, med (q1; q3)	56.5 (20.0; 186.5)	63.0 (21.0; 345.0)	59.5 (27.5 132.0)	0.898
ALAT U/L, med (q1; q3)	45.0 (26.0; 140.0)	37.0 (23.7; 162.0)	50.0 (19.0; 162.0)	0.335
GGT U/L, med (q1; q3)	308.5 (38.5; 996.5)	601.0 (43.0; 1067.0)	252.0 (36.0; 648.5)	0.327
ALP U/L, med (q1; q3)	197.0 (82.5; 492.5)	171.5 (83.2; 397.2)	243.0 (81.5; 575.5)	0.657
Amylase U/L, med (q1; q3)	60.0 (36.0; 93.5)	61.0 (39.0; 82.7)	60.0 (35.0; 101.5)	0.795
Lipase U/L, med (q1; q3)	40.5 (18.5; 105.5)	59.5 (23.2; 240.2)	31.5 (10.2; 105.5)	0.316
Total cholesterol mg/dL, med (q1; q3)	191.0 (122.7; 214.2)	191.2 (128.5; 242.5)	192.0 (108.5; 207.5)	0.598
HDL-cholesterol mg/dL, med (q1; q3)	44.2 (33.1; 50.2)	45.0 (31.7; 55.8)	42.6 (33.7; 45.7)	0.412
Triglycerides mg/dL, med (q1; q3)	112.0 (94.0; 223.0)	100.5 (88.0; 129.7)	135.0 (97.0; 288.0)	0.191
Albumin g/dL, med (q1; q3)	3.9 (3.5; 4.2)	4.1 (3.5; 4.2)	3.9 (3.2; 4.1)	0.400
CA 19-9 U/ mL, med (q1; q3)	907.9 (63.4; 11273.2)	550.0 (18.7; 11195.7)	1729.8 (75.4; 11505.5)	0.988
CA 125 U/ mL, med (q1; q3)	61.4 (24.4; 145.7)	33.5 (18.2; 119.3)	104.3 (48.3; 267.8)	0.090

***Abbreviations**: ALAT - alanine aminotransferase; ALP - alkaline phosphatase; ASAT - aspartate aminotransferase; CA - cancer antigen; CYFRA 21-1 - Cytokeratin 19-fragments; CRP – C-reactive protein; ESR – erythrocyte sedimentation rate; HDL - high-density lipoprotein; GGT - gamma-glutamyl transferase; OPN - osteopontin; HE4 - human epididymis protein 4.*
p-value <0.05 was considered statistically significant.

Table S4. Comparative analysis of the parameters analyzed in the patients with pancreatic cancer included in study in relation to low versus respectively high HE-4 levels in relation to the proposed cut-off (extended data compared to those presented in the article).

**Parameter**	**Cases** n=60	**Low HE4** < 0.29ng/mL n1=30	**High HE4** > 0.29ng/mL n2=30	**p-value**
Gender, M/F (%M)	26/ 34 (43.3%)	10/20 (33.3%)	16 (53.3%)	0.118
Age, years med (q1; q3)	69.5 (64.2; 76.0)	68.0 (55.7; 72.5)	73.5 (65.7; 77.0)	0.030
BMI, kg/m^2 med (q1; q3)	24.7 (21.9; 28.0)	25.9 (21.9; 29.2)	24.2 (21.8; 27.1)	0.283
Smoke, n (%)	9 (15.0%)	6 (20.0%)	3 (10.0%)	0.302
Alcohol, n (%)	15 (25.0%)	8 (26.7%)	7 (23.3%)	0.825
Meat-rich diet, n (%)	20 (33.3%)	13 (43.3%)	7 (23.3%)	0.140
Sweet-rich diet, n (%)	16 (26.7%)	8 (26.7%)	8 (26.7%)	0.934
High-fat diet, n (%)	13 (21.7%)	5 (16.7%)	8 (26.7%)	0.308
Abdominal pain, n (%)	33 (55.0%)	16 (53.3%)	17 (56.7%)	0.915
Heartburn, n (%)	18 (30.0%)	6 (20.0%)	12 (40.0%)	0.066
Vomiting, n (%)	19 (31.7%)	6 (20.0%)	13 (43.3%)	0.046
Nausea, n (%)	22 (36.7%)	10 (33.3%)	13 (43.3%)	0.424
Jaundice, n (%)	18 (30.0%)	10 (33.3%)	8 (26.7%)	0.630
Weight loss, n (%)	41 (68,3%)	19 (63.3%)	22 (73.3%)	0.340
CYFRA21-1, pg/mL med (q1; q3)	0.24 (0.13; 0.42)	0.17 (0.11; 0.28)	0.37 (015; 1.94)	0.002
OPN, ng/mL med (q1; q3)	3.37 (1.84; 9.12)	2.24 (1.43; 3.99)	6.60 (2.29; 13.64)	0.001
HE4, ng/mL med (q1; q3)	0.29 (0.15; 0.69)	0.15 (0.10; 0.19)	0.65 (0.37; 10.80)	<0.001
Leucocytes/ µL, med (q1; q3)	8100 (6675; 10975)	7025 (5535; 9527)	9940 (7330; 11965)	0.009
Neutrophils/ µL, med (q1; q3)	5570 (4425; 8040)	4960 (4247; 6372)	6780 (5150; 9165)	0.029
Lymphocytes/ µL, med (q1; q3)	1610 (1145; 2137)	1565 (1175; 1925)	1705 (967; 2247)	0.658
Hemoglobin g/dL, med (q1; q3)	12.4 (11.3; 13.7)	12.9 (11.9; 13.8)	11.8 (10.1; 13.2)	0.027
Thrombocytes/ µL, med (q1; q3)	280.0 (205.5; 344.5)	270.5 (201.5; 320.7)	294.0 (237.0; 372.0)	0.158
ESR mm/, med (q1; q3)	31.0 (20.7; 63.7)	50.0 (20.0; 106.0)	29.0 (21.0; 44.5)	0.324
CRP mg/dL, med (q1; q3)	14.5 (4.9; 86.0)	9.9 (3.2; 72.1)	22.9 (5.2; 126.5)	0.486
Bilirubin, mg/dL med (q1; q3)	1.4 (0.5; 7.4)	0.8 (0.5; 8.2)	2.3 (0.6; 7.4)	0.449
Creatinine, mg/dL med (q1; q3)	0.7 (0.6; 0.9)	0.6 (0.5; 0.7)	0.9 (0.7; 1.0)	<0.001
Uric acid, mg/dL med (q1; q3)	4.4 (3.7; 5.7)	3.9 (3.5; 4.7)	5.5 (4.0; 7.4)	0.016
ASAT U/L, med (q1; q3)	56.5 (20.0; 186.5)	39.0 (24.5; 173.0)	58.0 (26.0; 123.0)	0.323
ALAT U/L, med (q1; q3)	45.0 (26.0; 140.0)	59.0 (20.0; 364.5)	50.0 (21.0; 125.0)	0.399
GGT U/L, med (q1; q3)	308.5 (38.5; 996.5)	395.0 (33.0; 1067.0)	293.0 (52.5; 669.0)	0.657
ALP U/L, med (q1; q3)	197.0 (82.5; 492.5)	207.0 (82.5; 373.2)	168.0 (82.0; 577.0)	0.895
Amylase U/L, med (q1; q3)	60.0 (36.0; 93.5)	60.0 (37.0; 110.0)	60.0 (35.7; 93.2)	0.521
Lipase U/L, med (q1; q3)	40.5 (18.5; 105.5)	67.0 (20.0; 167.0)	31.5 (15.5; 97.5)	0.396
Total cholesterol mg/dL, med (q1; q3)	191.0 (122.7; 214.2)	204.0 (155.5; 249.2)	163.5 (117.7; 202.5)	0.114
HDL-cholesterol mg/dL, med (q1; q3)	44.2 (33.1; 50.2)	45.6 (28.5; 69.2)	43.6 (34.3; 46.0)	0.570
Triglycerides mg/dL, med (q1; q3)	112.0 (94.0; 223.0)	98.5 (79.5; 170.5)	114.0 (95.5; 240.5)	0.353
Albumin g/dL, med (q1; q3)	3.9 (3.5; 4.2)	4.1 (3.6; 4.3)	3.9 (3.1; 4.1)	0.082
CA 19-9 U/ mL, med (q1; q3)	907.9 (63.4; 11273.2)	260.3 (15.4; 3298.1)	3610.0 (168.1; 31335.2)	0.041
CA 125 U/ mL, med (q1; q3)	61.4 (24.4; 145.7)	45.4 (17.3; 83.2)	137.6(41.8; 442.3)	0.022

Abbreviations: *ALAT - alanine aminotransferase; ALP - alkaline phosphatase; ASAT - aspartate aminotransferase; CA - cancer antigen; CYFRA 21-1 - Cytokeratin 19-fragments; CRP – C-reactive protein; ESR – erythrocyte sedimentation rate; HDL - high-density lipoprotein; GGT - gamma-glutamyl transferase; OPN - osteopontin; HE4 - human epididymis protein 4.*
p-value <0.05 was considered statistically significant.
